# Dietary toxicity of soluble and insoluble molybdenum to northern bobwhite quail (*Colinus virginianus*)

**DOI:** 10.1007/s10646-015-1587-5

**Published:** 2015-12-12

**Authors:** Jennifer M. Stafford, Charles E. Lambert, Justin A. Zyskowski, Cheryl L. Engfehr, Oscar J. Fletcher, Shanna L. Clark, Asheesh Tiwary, Cynthia M. Gulde, Bradley E. Sample

**Affiliations:** Carolina Research Center, Smithers Viscient, Snow Camp, NC 27349 USA; McDaniel Lambert Inc. (an Intrinsik Company), 1608 Pacific Ave, Suite 201, Venice, CA 90291 USA; Diagnostic Center for Population and Animal Health, College of Veterinary Medicine, Michigan State University, 4125 Beaumont Rd, Lansing, MI 48910 USA; Department of Population Health & Pathobiology, College of Veterinary Medicine, NC State University, 1060 William Moore Dr., Raleigh, NC 27607 USA; Chevron Energy Technology Company, 1200 Smith Street, Houston, TX 77002 USA; Chevron EMC, 116 Inverness Drive East, Suite 207, Englewood, CO 80112 USA; Ecological Risk, Inc., 15036 Magno Ct., Rancho Murieta, CA 95683 USA

**Keywords:** Avian, Dietary toxicity, Molybdenum, NOAEC

## Abstract

Limited data are available on the effects of molybdenum (Mo) on avian wildlife, which impairs evaluation of ecological exposure and risk. While Mo is an essential trace nutrient in birds, little is known of its toxicity to birds exposed to molybdenum disulfide (MoS_2_), the predominant form found in molybdenite ore. The chemical form and bioavailability of Mo is important in determining its toxicity. Avian toxicity tests typically involve a soluble form of Mo, such as sodium molybdate dihydrate (SMD, Na_2_MoO_4_·2H_2_O); however MoS_2_ is generally insoluble, with low bioaccessibility under most environmental conditions. The current study monitored survival and general health (body weight and food consumption) of 9-day old northern bobwhite exposed to soluble Mo (SMD) and ore-related Mo (MoS_2_) in their diet for 30 days. Toxicity and bioavailability (e.g. tissue distribution) of the two Mo forms were compared. Histopathology evaluations and serum, kidney, liver, and bone tissue sample analyses were conducted. Copper, a nutrient integrally associated with Mo toxicity, was also measured in the diet and tissue. No treatment-related mortality occurred and no treatment-related lesions were recorded for either Mo form. Tissue analyses detected increased Mo concentrations in serum, kidney, liver, and bone tissues following exposure to SMD, with decreasing concentrations following a post-exposure period. For the soluble form, a No-Observed-Adverse-Effect Concentration (NOAEC) of 1200 mg Mo as SMD/kg feed (134 mg SMD/kg body weight/day) was identified based on body weight and food consumption. No adverse effects were observed in birds exposed to MoS_2_ at the maximum dose of 5000 mg MoS_2_/kg feed (545 mg MoS_2_/kg body weight/day). These results show that effects associated with MoS_2_, the more environmentally prevalent and less bioavailable Mo form, are much less than those observed for SMD. These data should support more realistic representations of exposure and risks to avian receptors from environmental Mo.

## Introduction

Molybdenum (Mo) is widely distributed in the environment, with concentrations varying with geology (Adriano 1986; Chappell and Petersen 1976). Typical, natural concentrations of Mo in soils average 1–2 mg/kg and may range from trace concentrations to 40 mg/kg or greater (Adriano 1986; Kubota 1977; USGS 2015). Mo residues tend to be elevated in plants and soils near Mo mining and reclamation sites and fossil-fuel power plants. However, Mo concentrations are usually lower in fish and wildlife than in terrestrial plants (Eisler 1989). Concentrations in soils in the vicinity of a Mo mine in Mongolia ranged from 2 to 19 times greater than that in background soils, with concentrations at the tailings impoundment being 60–456 times greater than background (Battogtokh et al. 2014). At the Benda Mine in British Columbia, Canada, Taylor and McKee (2003) report mean Mo concentrations ranging from 61 to 275 mg/kg for vegetation growing in mine tailings with Mo concentrations of 50–140 mg/kg. At another mine in British Columbia, Mo concentrations in soil ranged from 1.2 to 139 mg/kg, with concentrations in grass and legumes ranging from 2 to 341 mg/kg and 2 to 1030 mg/kg, respectively (Jones 1994).

Limited data are available on the effects of Mo on avian species. Since Mo is an essential trace mineral in birds, the majority of Mo avian studies identified in the literature are dietary studies of soluble forms of Mo [e.g. sodium molybdate dihydrate (SMD)] on domestic species (chickens and turkeys) conducted from the 1950s through the 1970s (Kratzer 1952; Arthur et al. 1958; Lepore et al. 1961; Lepore and Miller 1965; Friberg et al. 1975; Kienholz 1977). Birds appear to have a lower biological requirement for Mo [up to 6 parts per million (ppm); Eisler 1989] than most mammalian species, but a higher resistance to excess Mo (Eisler 1989; Davies et al. 1960). Mortality among chickens is observed at dietary concentrations of 6000 ppm Mo as molybdate (Eisler 1989). Concentrations of dietary SMD of 6 ppm or less are reported to improve weight gain of growing chicks (Eisler 1989). Mo toxicosis in most animals is largely secondary to copper (Cu) deficiency, and this appears to be the case for birds as well (NRC 2005). Sulfur also plays a role in Mo toxicity because of the potential formation of thiomolybdates in the stomach which reduce Cu bioavailability (NRC 2005). Growth (weight gain) effects in birds generally are evident at approximately 500 ppm Mo as molybdate in the diet, lower if copper is deficient (Davies et al. 1960). Growth depression in chicks has been observed in chicks at 200 ppm (Kratzer 1952). Based on these data, NRC (2005) suggested a maximum tolerable level for poultry of 100 ppm in feed. Soluble forms of Mo (such as SMD) appear to be more toxic to birds than insoluble forms such as Mo disulfide (Davies et al. 1960). Available data for chickens and turkeys suggests that birds are relatively resistant to excess soluble Mo, with effects dependent on the ratios of Mo, copper, and sulfate in the diet, however, there is a wide range of effects noted and disagreement in the literature as to what the avian Mo lowest no observed adverse effect levels (NOAELs) are (NRC 2005).

Molybdenum disulfide (MoS_2_) is the form of Mo most commonly found in molybdenite ore bodies in North America; therefore the toxicity of this form of Mo is of interest with respect to potential ecological exposures to soils around Mo mining operations. No data are currently available on the toxicity of SMD or the less soluble MoS_2_ in avian wildlife species. To fill this data gap, the present study exposed the northern bobwhite quail (*Colinus virginianus*), a commonly used U.S. Environmental Protection Agency (EPA) model in avian toxicity studies, to either SMD or MoS_2_ in their diet for 30 days. The birds were evaluated for changes in body weight, food consumption, blood Mo and Cu concentrations, histopathology, and other signs of toxicity. To assess the reversibility of potential effects, specific endpoints also were evaluated following a 5 day recovery period, during which birds received untreated feed.

## Materials and methods

### Test species

Wild-type northern bobwhite quail were obtained from Shady Knoll Gamebirds in Asheboro, NC, USA. Birds were acclimated for 7 days, and evaluated visually for signs of illness, disease, or mortality. Each bird was affixed with a Monel 1005-3 wing tag imprinted with a unique 4-digit ID number. All 108 birds were confirmed to be healthy at test initiation. Birds were 9 days old at experimental start; at this age the plumage of bobwhite chicks is not sexually dimorphic. Therefore, chick sex was not considered during selection for study use or the assignment to test cages. However, as chicks were terminated and necropsied during the study, the sex of each was determined. Ultimately, the study included 46 females and 62 males.

### Test conditions

Birds were housed in thermostatically-controlled cages measuring 81 cm wide × 91 cm deep × 25 cm high, with brooder heaters positioned above one end of each cage. At this age quail must be housed in groups to support natural coveying, thermoregulatory, and feed- and water-finding behaviors, which are very difficult for singly-housed chicks to accomplish. Because the birds required group housing, chicks were housed in groups of 12 per cage with one cage randomly assigned to each test group.

Room temperatures ranged from 22 to 30 degrees Celsius (°C). Temperatures directly under brooder heat ranged from 37 to 39 °C while birds were 0–7 days old, 33–38 °C while birds were 8–14 days old, and 26–35 °C as birds aged 14 days. Humidity ranged from 35 to 69 %. Ventilation within the study room maintained at least 15.2 air exchanges per hour. A light schedule of 14:10 h light:dark cycle was provided with two 15 min transition periods. Average light intensity was approximately 37.2 foot-candles. Birds were supplied with water from a deep well that was analyzed for the presence of pesticides, polychlorinated biphenyls, and metals. Concentrations of all of these compounds were below detection limits. Mo also was not detected in the water sample [at the limit of quantitation (LOQ) of 0.0400 ppm].

All birds were observed twice daily for the initial 10 days of the study, and once daily for the remainder of the study. Birds were monitored for general condition, mortality, and signs of intoxication.

### Test diet

Birds were fed a 28 % protein diet of Bartlett Gamebird Starter Crumbles (Bartlett Milling Company LP, Statesville NC) ad libitum during the acclimation, exposure and recovery study periods. The same lot of starter feed was used during each study period, and to prepare the test diets. Background concentrations of Mo in the basal feed, prior to incorporation of either SMD or MoS_2_, were not detectable (LOQ 5.40 ppm). SMD (Na_2_MoO_4_*2H_2_O), also known as molybdic acid disodium salt, was obtained from AAA Mo Products, Inc., Broomfield, Colorado, USA. SMD used in this study was reported to have a purity level of 99.9 % SMD. MoS_2_, was also obtained from AAA Mo Products, Inc., with a purity level of 98.85 % Mo disulfide. Control diets were prepared by adding approximately 10 ml acetone to 8.0 kg basal feed, and mixing the combination in a Max Sphere kinematic-motion mixer for 10 min. The resulting diet was stored in a sealed, labeled container at ambient conditions. Treated diet mix was prepared by adding finely ground SMD or MoS_2_ to a pre-weighed aliquot of basal feed. Approximately 10 ml acetone was used to rinse the vessel into which the test article was weighed, adding the rinsate to the feed. A total of 8 kg of each test diet was prepared, with each diet mixed in two 4 kg aliquots in glass containers. The two aliquots were combined in the same sealed, labeled storage container when complete. The prepared test diets were stored at ambient conditions. Dietary treatment concentrations were calculated based on the weight of Mo as follows: 0, 420, 700, 1200, 2000 and 3000 mg Mo (as SMD)/kg feed, and 700, 3000 and 5000 mg Mo (as MoS_2_)/kg feed. Note that these dietary concentrations are generally high relative to concentrations measured in biota in general (<0.1–560 mg/kg dry weight; Eisler 1989) and plants at mine sites (2–1030 mg/kg; Jones 1994; Taylor and McKee 2003). In order to verify proper dietary concentrations of the SMD and MoS_2_ prior to bird consumption, test diets were analyzed via inductively coupled plasma mass spectroscopy (ICP-MS). Test diet concentration, homogeneity and ambient stability of each substance were confirmed in the test diet prior to administering them to the test animals.

### Exposure regimen

Prior to treatment, each bird was identified by a uniquely numbered wing tag, weighed, and randomly assigned to one of nine test cages. Twelve birds were assigned to each cage. The cages were randomly assigned to a unique test group using the random number generator using Microsoft^®^ Excel software. Each cage received one of the following test groups (dietary exposure concentrations): control, 420, 700, 1200, 2000, and 3000 mg Mo as SMD/kg feed, and 700, 2000, and 5000 mg Mo as MoS_2_/kg feed. The birds in each group received their assigned, fixed-concentration test diet for 30 days (without adjustment on a body weight basis) and were observed daily. At the end of the exposure period (day 30), six birds from each cage were terminated. The remaining six birds from each cage entered a post-exposure period, or recovery period, during which birds received clean (untreated) feed for an additional 5 days before they were terminated (day 35).

### Body weight

Body weight was measured for each bird at least three times during the study: days 0, 15, and 30. Body weight also was measured at the end of the post-exposure period (day 35) for those birds that were maintained to study termination. Measured body weights were used to calculate proportional change in weight, as [body weight at interval 2 (in grams, g) − body weight at interval 1 (g)]/[body weight at interval 1 (g)].

### Feed consumption

Feed consumption per cage was measured daily. Spillage was captured and was accounted for in feed measurements. Daily cage feed consumption was divided by the number of birds in each cage to calculate consumption/bird/day. Average daily feed consumption, along with the calculated mean body weight per bird, was used to estimate the mean daily exposure per bird [mg active ingredient (a.i.)/kg body weight/day].

### Necropsy, histopathology and tissue sampling

Information on lesions from Mo in birds is generally lacking. Therefore several different potential target organs were evaluated to assess developmental and survival-related implications of the possibility that Mo may be preferentially accumulated similar to other heavy metals. During the day 30 and 35 sacrifice events, blood and tissue samples were collected and all sacrificed birds underwent gross necropsy. Any unusual lesion was collected and preserved for histological evaluation. Collected blood was spun down to extract the serum, which was stored in royal blue top (EDTA) tubes, frozen and then shipped to Michigan State University (MSU) for Mo and Cu content analysis. One liver lobe, one kidney lobe, and one femur and tibiotarsus were collected from each bird and frozen. These samples also were sent to MSU for Mo and Cu content analyses. A second liver lobe, kidney lobe and femur and tibiotarsus, along with one lung, the heart, bursa of fabricius, brain, thymus glands, and ovary/testes were collected from each bird, placed in 10 % formalin, and sent to North Carolina State University (NCSU) for histological evaluation. The bones sent to NCSU also underwent morphometric analysis for refined assessment of differences in the thickness of the periosteum and cortical bone.

### Analytical method for detection of copper (Cu) and molybdenum (Mo) in feed and tissue samples

Copper and Mo analyses were performed by ICP-MS by the Diagnostic Center for Population and Animal Health in the College of Veterinary Medicine at MSU.

Feed samples (moisture content ranging from 7 to 9 %) and organ tissue samples (sectioned to 1-g pieces) were dried overnight in a 75-°C oven. Moisture content in liver samples ranged from 75 to 94 %, moisture content in kidney samples ranged from 86 to 99 %, and moisture content in bone samples ranged from 19 to 64 %. Dry feeds and dried tissues were weighed out and digested overnight in a 95 °C oven in individually sealed vessels with 1 ml nitric acid:100 mg feed or dry tissue ratio. The resulting solution was diluted with 18 milliohm (MΩ) water to a final mass of 25 g. Feed sample preparations were diluted further with 18 MΩ water to lower the concentration of the analytes in the diluted samples to within the calibration range.

Two hundred microliters (µL) of each digest, as well as all serum/blood samples, were pipetted and diluted with 5 mL of a solution containing 0.5 % EDTA and Triton X-100, 1 % ammonia hydroxide, 2 % propanol and 20 parts per billion (ppb) of scandium, rhodium, indium and bismuth as internal standards. An Agilent 7500ce ICP-MS was used for the analysis. The ICP-MS was tuned to yield a minimum of 5000 cps sensitivity for 1 ppb yttrium (mass 89), less than 1.0 % oxide level as determined by the 156/140 mass ratio and less than 2.0 % double-charged ions as determined by the 70/140 mass ratio. Each element was calibrated using a 4-point linear curve of the analyte:internal standard response ratio. Three modes were used to minimize the spectral interferences for the analysis.

### Data analysis

For each test group, proportional body weight change, serum and tissue Cu and Mo concentrations, and bone morphometrics were evaluated for differences from the control using analysis of variance (ANOVA) followed by Dunnett’s post hoc test. The day 30 serum residue data set contained one sample of insufficient volume for analysis, which was therefore excluded, and the resulting data set was not normally distributed; therefore this data set was analyzed via Steel’s many-one rank test (non-parametric analysis for unequal replicates). Feed consumption per quail was reported as an average per cage and therefore not statistically analyzed. Statistical analyses were performed in TOXSTAT^®^ v3.5, excluding bone morphometry which was evaluated using SAS v9.3.

## Results

### Feed analysis

Mo recoveries from prepared diet samples are summarized in Table 1. Mean measured recoveries ranged from 97.9 to 116.6 % of nominal, with coefficients of variation ranging from 1.2 to 4.4 %. Dietary samples measured at the end of the study yielded recoveries ranging from 95.6 to 101.6 % of Mean day 0 recoveries, verifying that acceptable stability of the feed Mo content was maintained throughout the study.

**Table 1:** Analytical Mo and Cu recoveries in feed used to expose northern bobwhite (*Colinus virginianus*) during a 30-day dietary toxicity test

Treatment concentration (mg Mo/kg feed dry weight)	Measured feed concentration (and standard deviation) mg/kg feed dry weight		Measured Mo concentration as % of target	Stability of test substance at end of study as % of day 0	Average Mo exposure (mg Mo/kg body weight/day^a^)
	Mo	Cu			
Control (background)	2.2 (0.11)	31.2 (4.2)	NA	NA	0
420	423.2 (10.5)	27.3 (2.4)	101 %	99.7 %	45.3
700	702.9 (9.2)	28.6 (3.3)	100 %	NA	73.6
1200	1235 (12.2)	28.3 (1.6)	103 %	NA	134
2000	2231 (29.9)	31 (3.8)	112 %	NA	253
3000	3056 (134.0)	26.4 (4.2)	102 %	101 %	362
700	727.4 (8.4)	27.1 (3.4)	104 %	95.6 %	75.4
2000	2219 (36.2)	28.8 (3.1)	111 %	NA	218
5000	5266 (219.0)	27.6 (3.2)	105 %	101 %	545

*NA* not analyzed or not applicable

^a^Calculated from actual feed consumption rates and measured body weights collected during the study, not dietary analysis results; presented here for comparison of the exposure presented and the exposure experienced

Dietary Cu varied little among treatments, ranging from 31.2 mg/kg in the control to 26.4 in the 3000 ppm Mo as SMD test group (Table 1). The dietary copper requirement for bobwhite quail ranges from 6.6 to 7.9 mg/kg in feed (Ferket 1989), with no toxicity observed in birds at dietary concentrations less than 250 mg/kg (NRC 2005). Therefore, although dietary copper in all treatments exceeded the minimum requirement, it was not sufficiently elevated to contribute to toxicity.

### Body weight

**Fig. 1:**
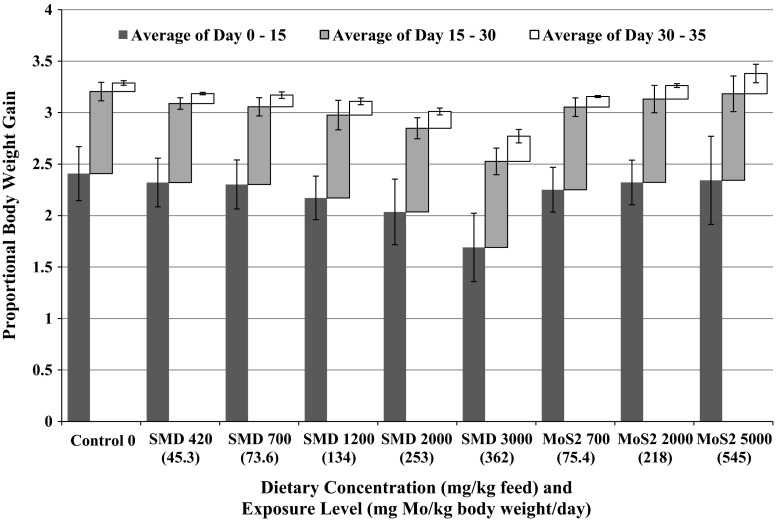
Mean (standard deviation) proportional body weight change of northern bobwhite (*Colinus virginianus*) exposed to dietary Mo for 30 days. Weight gain significantly reduced in the 2000 and 3000 ppm Mo as SMD test groups for Day 0–15 and for the entire exposure period (Day 0–30). No growth reduction observed for any other test group or interval

Body weight data are summarized in Fig. 1. Body weights were homogenous, normally distributed, and not significantly different among groups at treatment initiation. Relative to the control, proportional body weight gain was significantly reduced during the first half of exposure (day 0–15) and for the entire exposure period (day 0–30) in the 2000 and 3000 ppm Mo as SMD test groups. No differences in proportional weight gain were observed for any of the MoS_2_ test groups relative to the control group during the exposure period. During the post-exposure period (days 30–35), neither the SMD nor MoS_2_ groups were significantly different in proportional body weight gain. By the end of the recovery period (day 35), proportional weight gain in the test groups for either form of Mo were not significantly different from the control group.

Mean (standard deviation) proportional body weight change of northern bobwhite (*Colinus virginianus*) exposed to dietary Mo for 30 days. Weight gain significantly reduced in the 2000 and 3000 ppm Mo as SMD test groups for day 0–15 and for the entire exposure period (day 0–30). No growth reduction observed for any other test group or interval.

### Feed consumption

For each test group, body weight measurements collected during the 30-day exposure period were averaged. For each test group, the average feed consumed per bird per day also was calculated (using test group consumption rates divided by the appropriate number of quail present) for both the exposure and post-exposure period (Table 2); these values were used to calculate per-bird exposure concentrations. Based on measured body weights and the average quantity of feed consumed, realized Mo exposure concentrations for each group were 0, 45.3, 73.6, 134, 253 and 362 mg Mo (as SMD)/kg body weight/day, and 75.4, 218 and 545 mg Mo (as MoS_2_)/kg body weight/day (Table 1). Due to disturbance to the birds caused by body weight collection and sacrifice at each interval, the actual time available to the birds to feed during days 31 and 35 are not quantifiable. Therefore, the value of feed consumed per bird per day could not be calculated for these 2 days, and as such have been omitted from statistical analysis and graphical summary of the feed consumption data. As shown in Table 2, feed consumption rates at 3000 ppm SMD were decreased slightly. Figure 1 above shows a corresponding decrease in body weight gain at 3000 ppm SMD.

**Table 2:** Mean feed consumption (g/bird/day) measured during the northern bobwhite (*Colinus virginianus*) 30-day dietary toxicity test with Mo

Treatment concentration (mg Mo/kg feed dry weight)	Exposure period					Post-exposure (days 31–35)^b^	Average total feed consumption/bird/day (g)^c^	Number of males and females per cage (males/females)
	Week 1	Week 2	Week 3	Week 4	Week 5 (days 29 and 30)			
Control	5.7 (0.86)^a^	9.0 (1.1)	12 (1.6)	14 (1.8)	13 (1.8)	16 (1.0)	10 (3.4)	8/4
Sodium molybdate dihydrate (SMD, Na_2_MoO_4_·2H_2_O)								
420	5.8 (0.84)	8.6 (1.1)	11 (1.4)	13 (1.9)	12 (2.1)	14 (3.3)	9.8 (3.1)	5/7
700	5.9 (0.84)	5.5 (1.2)	11 (1.5)	13 (2.6)	11 (1.5)	14 (0.8)	9.5 (3.0)	9/3
1200	8.4 (0.8)	8.3 (0.8)	11 (1.0)	13 (1.2)	12 (2.7)	14 (0.4)	9.7 (3.1)	8/4
2000	5.1 (0.88)	8.7 (1.3)	12 (1.8)	15 (2.1)	13 (1.3)	16 (1.9)	10 (4.0)	5/7
3000	5.0 (0.66)	6.6 (1.2)	9.5 (1.5)	12 (2.1)	11 (3.5)	15 (1.1)	8.4 (3.0)	7/5
Molybdenum disulfide (MoS_2_)								
700	5.6 (0.77)	9.0 (1.1)	11.5 (1.3)	14.0 (1.3)	11.7 (1.6)	15 (1.1)	10 (3.3)	8/4
2000	5.6 (0.73)	8.6 (1.3)	11.0 (1.1)	13.8 (1.7)	12.6 (0.8)	14 (0.5)	9.9 (3.3)	8/4
5000	5.8 (0.65)	8.6 (0.79)	11.4 (1.2)	13.2 (1.3)	12.1 (1.4)	15 (1.5)	9.9 (3.0)	4/8

^a^Standard deviation in parenthesis

^b^Note, due to disturbance to the birds caused by body weight collection and selection of birds for sacrifice at each interval, the actual time available to the birds to feed during days 31 and 35 are not quantifiable. Therefore, the value of feed consumed per bird per day could not be calculated for these 2 days, and as such have been omitted from statistical analysis and graphical summary of the feed consumption data

^c^Data not analyzed statistically since the feed consumption for each group was combined for all animals in a test group

### Necropsy, histopathology and morphometric analysis

Neither treatment-related lesions nor any other abnormal gross findings were reported in the control or any of the SMD or MoS_2_ test groups during the day 30 and 35 necropsies. Histopathology evaluations of lung, heart, bursa of fabricius, brain, thymus, ovary and testes samples identified various incidental, infrequent, non-treatment related lesions (e.g. occasional bursa of fabricius cysts, focal hemorrhage in heart, and proliferative response and/or thrombocytes in lung). Lesions were scored and compared (data not shown), and were not present in a dose-dependent or recurring pattern.

Because twenty of the 108 quail used in the study were noted at gross necropsy/histopathology examination as having possible thickening of the periosteum and cortical bone, the femur and tibiotarsus of these 20 birds were evaluated further via morphometric analysis. To facilitate comparison across all groups, additional bone samples from which measurable periosteum was present also were included in the morphometric analysis, but samples that did not present measurable periosteum were not further evaluated. To evaluate whether the possible visual anomalies were treatment-related, morphometric measurements (data not shown) were compared via ANOVA in SAS (SAS 2010). No statistically significant differences between the control and any test/comparator groups were observed in average periosteal width at day 30 (*p* = 0.5984) or day 35 (*p* = 0.4721), or in average cortical bone width at day 30 (*p* = 0.5485) or day 35 (*p* = 0.0470; difference not between control and test groups), indicating that the variances in these parameters observed during the visual histological assessments were not treatment-related.

### Serum Mo and Cu concentrations

Serum Mo concentrations increased significantly at 420, 700 and 2000 ppm Mo as SMD relative to the control group at the end of the exposure period (day 30; Table 3). However, serum Mo increases appear to be lowest for the highest SMD test group (3000 ppm Mo as SMD). This may be related to the relative decrease in feed consumption in this group and/or a point in time decrease at the time the group was sampled. Serum Mo concentrations for all MoS_2_ test groups were not significantly different from the control group at the end of the exposure period (day 30). Serum Mo concentrations declined by the end of the 5-day post-exposure period (day 35), but remained significantly higher in all SMD and MoS_2_ test groups relative to the control group at the end of the post-exposure period.

**Table 3:** Mean (standard deviation) Mo and Cu concentrations detected in serum, kidney, liver and bone tissues during the northern bobwhite (*Colinus virginianus*) 30-day dietary toxicity test

Treatment concentration (mg Mo/kg feed dry weight)	Serum		Kidney		Liver		Long bones (femur and tibiotarsus)	
	Day 30	Day 35	Day 30	Day 35	Day 30	Day 35	Day 30	Day 35
Mean Mo concentrations in serum and tissue samples (ppb or ng/g dry weight)								
Control	54.5 (16.9)	37.2 (5.3)	5899 (637.9)	6153 (1768)	6329 (1054)	6984 (308.8)	551.9 (240.6)	449.1 (89.21)
Sodium molybdate dihydrate (SMD; Na_2_MoO_4_·2H_2_O)								
420	1911^a^ (1026)	174.8^a^ (103.0)	9762 (6131)	5731 (2391)	11,530^a^ (1660)	7621 (778.4)	28,673^a^ (11,240)	9683^a^ (3805)
700	1821^a^ (1288)	139.2^a^ (29.4)	9722 (3493)	5110 (1624)	11,890^a^ (3312)	7373 (1055)	28,900^a^ (12,320)	9680^a^ (3295)
1200	1026^a^ (616.4)	157.5^a^ (51.5)	12,440^a^ (2640)	8304 (1058)	9152^a^ (1904)	6809 (1607)	31,780^a^ (7993)	17,870^a^ (5723)
2000	1815^a^ (644.4)	203.7^a^ (23.0)	17,340^a^ (2713)	8842^a^ (675.7)	13030^a^ (3001)	6633 (1162)	57,550^a^ (7295)	21,350^a^ (3267)
3000	756.7^a^ (188.2)	247.2^a^ (58.0)	12,390^a^ (3114)	6197 (2187)	10980^a^ (968)	8248 (1713)	57,750^a^ (7932)	27,995^a^ (3931)
Molybdenum disulfide (MoS_2_)								
700	84.6 (23.6)	74.8^a^ (12.56)	4942 (2623)	4891 (2259)	5664 (628)	5494 (276)	457.1 (60.55)	417.5 (72.5)
2000	97.4 (33.4)	74.23^a^ (4.4)	8463 (1132)	7223 (1910)	5082 (712.7)	5361 (589.8)	668.1 (85.0)	412.3 (46.2)
5000	71.9 (13.5)	73.0^a^ (10.8)	6659 (1995)	3489 (2311)	4576 (755.7)	6259 (543.1)	1278^a^ (362.8)	531.0 (112.8)
Mean Cu concentrations in serum and tissue samples (ppb or ng/g dry weight)								
Control	0.16 (0.06)	0.19 (0.07)	12.3 (1.5)	11.9 (4.1)	20.6 (3.4)	23.1 (3.1)	0.86 (0.23)	0.81 (0.21)
Sodium molybdate dihydrate (SMD; Na_2_MoO_4_·2H_2_O)								
420	0.19 (0.08)	0.17 (0.05)	8.4 (4.9)	9.0 (4.5)	21.0 (3.5)	20.5 (1.5)	0.89 (0.13)	0.74 (0.07)
700	0.17 (0.06)	0.22 (0.13)	11.4 (6.8)	8.8 (2.6)	19.8 (2.2)	18.7 (1.4)	0.55 (0.26)	0.33 (0.28)
1200	0.15 (0.01)	0.16 (0.05)	16.7 (0.9)	15.8 (1.2)	17.2 (2.5)	18.6 (1.6)	1.22 (1.19)	1.34 (1.54)
2000	0.22 (0.07)	0.16 (0.04)	17.0 (3.2)	15.5 (1.4)	16.0^a^ (1.9)	15.9^a^ (2.2)	ND^b^	0.71 (0.91)
3000	0.22 (0.07)	0.21 (0.03)	11.2 (3.8)	7.5 (4.2)	15.6^a^ (1.4)	20 (6.5)	0.71 (0.13)	0.72 (0.12)
Molybdenum disulfide (MoS_2_)								
700	0.21 (0.04)	0.20 (0.05)	8.1 (6.1)	8.9 (5.4)	17.5 (2.7)	18.1^a^ (1.5)	0.71 (0.14)	0.67 (0.09)
2000	0.23 (0.04)	0.15 (0.02)	12.3 (2.3)	12.5 (3.1)	16.5 (2.9)	16.3^a^ (1.8)	0.63 (0.51)	0.42 (0.31)
5000	0.19 (0.05)	0.19 (0.08)	10.0 (3.7)	6.3 (4.2)	15.4^a^ (2.6)	18.8 (2.1)	0.68 (1.30)	1.57 (2.33)

^a^Significantly different from control based on ANOVA followed by Dunnett’s tests *p* < 0.05

^b^Cu below detection limit in all samples

No significant differences in serum Cu concentrations were observed for any SMD or MoS_2_ treatment for either the exposure period (through day 30) or the post-exposure period (day 35; Table 3).

### Liver, kidney and long bone analyses

Statistically significant increases in liver Mo concentrations were detected at 420–3000 ppm Mo as SMD (45.3–362 mg Mo/kg body weight/day exposure concentrations, respectively) compared to the control at the end of the exposure period (day 30). However, by the end of the 5-day post-exposure period (day 35), all groups returned to near-control concentrations, with no statistically significant differences among groups (Table 3). At the end of the 30 day exposure period, liver Cu was significantly reduced in the two highest SMD groups (2000 and 3000 ppm Mo as SMD) and in the highest MoS_2_ test group (5000 ppm Mo as MoS_2_). Post-exposure (day 35) liver Cu concentrations remained significantly reduced in the 2000 ppm but not the 3000 Mo as SMD group. Post exposure liver Cu was also significantly reduced in the 700 and 2000 ppm Mo as MoS_2_ group, but not in the 5000 ppm Mo group. However, because these mean post exposure liver Cu concentrations are equal to or greater than that observed at the end of the exposure period, these differences likely represent random variation, and not a treatment-related response.

Mo concentrations in kidneys increased at 1200, 2000 and 3000 ppm Mo as SMD compared to the control at the end of the exposure period (day 30). Kidney Mo concentrations decreased to near-control concentrations in 1200 and 3000 ppm Mo as SMD groups during the post-exposure period (day 35), but remained statistically higher at 2000 ppm Mo as SMD. No statistically significant differences in Mo concentrations were observed in kidney samples from any MoS_2_ group relative to the control (Table 3). Similarly, no significant differences in kidney Cu concentrations were observed for any SMD or MoS_2_ group relative to the control (Table 3).

Mo concentrations in long bones were significantly higher in all SMD test groups (420–3000 ppm) and the 5000 ppm MoS_2_ test group compared to the control at the end of the exposure period (day 30). Following the post-exposure period (day 35), bone Mo concentrations remained statistically higher than the control for all SMD groups, but returned to near-control concentrations in the 5000 ppm MoS_2_ group. No significant differences in long bone Cu concentrations were observed for any SMD or MoS_2_ group relative to the control (Table 3).

## Discussion

The effect of high dietary Mo intake depends on multiple factors, with copper and sulfur content of the diet being dominant (NRC 2005). Other factors that may influence Mo effects include (as summarized in Underwood 1977) the species and age of the animal; the amount and chemical form of the ingested Mo; and the inorganic sulfate and total sulfur content of the diet and its content of substances such as protein, cysteine, and methionine, capable of oxidation to sulfate in the body. This study tested two different forms of Mo: soluble SMD, a form commonly used in Mo toxicity studies, and highly insoluble MoS_2_, a form often associated with Mo ore and found in soils near molybdenite mining operations (Butler and Vanderwilt 1933; IMOA 2015). Growth (as measured by proportional body weight change) was reduced in the 2000 and 3000 ppm Mo as SMD treatments but recovered in the post exposure period and was not reduced in any other SMD test group or for any of the MoS_2_ test groups. This transient effect in proportional body weight gain among the two highest SMD treatment concentrations maybe partially due to food avoidance. Upon access to clean, untreated food, the rate of food consumption and body weight gain in birds previously exposed to the 2000 and 3000 ppm Mo as SMD test groups returned to near-control level within 5 days. Although some authors report effects on growth in birds at dietary concentrations as low as 200 mg Mo/kg diet (Eisler 1989), the finding of transient body weight gain depression and no mortality among northern bobwhite chicks when exposed to dietary concentrations of Mo as high as 5000 ppm SMD is consistent with previously published data for domesticated gallinaceous species, i.e. chickens and turkeys (Davies et al. 1960).

Mo is an essential trace nutrient in the diet of chicks and turkey poults (Underwood 1971, as cited in Eisler 1989). Adverse effects on growth have been reported for domestic birds at dietary Mo concentrations of 200 mg Mo/kg body weight/day (Eisler 1989). Although the present study in northern bobwhite quail demonstrated that effects on growth (as proportional body weight gain) occurred for the SMD formulation at dietary doses greater than 200 mg Mo/kg, these growth effects did not occur for the MoS_2_ formulation, including dietary doses greater than 200 mg Mo/kg (e.g., no effects on proportional body weight change were seen at 218 and 545 mg Mo/kg equivalent when Mo disulfide was incorporated in feed). Further, any growth-related effects identified for the SMD test groups were absent following the 5-day recovery period. Thus, the relatively quick elimination of Mo observed in mammalian species [e.g. half-lives in rats on the order of hours (Friberg and Lener 1986); complete Mo excretion guinea pigs within 7 days (Friberg et al. 1975)] also appears to be relevant in quail species. These findings suggests that (a) Mo disulfide does not result in the same effects on growth as SMD in diet, and (b) excretion kinetics should be considered (along with exposure time) when determining if an exposed bird population could experience growth effects, given that recovery occurred within 5 days of SMD diet exposure cessation. Since inorganic sulfate in the diet alleviates Mo toxicity in all known species by increasing urinary Mo excretion (Underwood 1977), the differential reversible effect on growth (produced by SMD at 2000 and 3000 ppm in diet, but not produced by MoS_2_) may be related to the presence of inorganic sulfate. Alternatively, the differences in observed effects between the two Mo forms may simply be a function of solubility and absorption rates; whereas SMD is readily soluble and absorbed, MoS_2_ is not.

Regardless of Mo bone deposition during exposure (through day 30), the post-exposure (day 35) results for SMD suggest that deposition is quickly reversible, although the refractory period of 5 days was not long enough to see a complete return to baseline. This study confirmed bone deposition of Mo if administered as SMD but not MoS_2_, clarifying previous literature that found dietary loadings of 2000 ppm induced a 100× increase in Mo content in domestic chicks tibia (Davies et al. 1960). Specifically, this study shows (Table 3) that SMD at all doses caused Mo deposition in the femur and tibiotarsus of quail chicks (100× increase in tibia Mo content at 3000 ppm). Deposition of Mo in bones for MoS_2_ occurred only at the highest dose of 5000 ppm.

Despite Mo deposition in bone, there were no treatment-dependent effects on periosteum thickness or cortex thickness of the femur or tibia samples analyzed at the end of the exposure (day 30) or study (day 35) period. Five days post-exposure, organ (liver and kidney) tissue Mo concentrations for all test, regardless of form, returned to near-control concentrations. In addition, examination of the lung, heart, bursa of fabrisicus, brain, thymus, ovaries and testes all were unremarkable and did not identify any treatment-related lesions.

Bioavailability in this study can be assessed based on the pattern of Mo concentrations in tissue samples at the end of the exposure and post-exposure periods. General patterns observed during this study were (a) increases in Mo concentrations in serum, kidney, liver, and bone during periods of continual dietary exposure to soluble Mo in the form of SMD (i.e., bioavailability of Mo as SMD), (b) marked decreases in these concentrations in as few as 5 days following removal of SMD from the diet, and (c) few significant increases in Mo concentrations in serum or tissues during period of continual dietary exposure to the insoluble, but environmentally prevalent Mo in the form of MoS_2_ (i.e., minimal bioavailability of Mo as MoS_2_).

This low bioavailability of MoS_2_ plays a significant role in the differences in effects on growth and uptake seen between the two forms of Mo evaluated in this study. In an unpublished range finding study (Drexler 2013, unpublished) conducted leading up to the study described herein, 2000 ppm each of SMD and MoS_2_ in feed were analyzed using an avian physiologically-based extraction test (Furman et al. 2006) to measure relative bioaccessibility.^1^ The method from Furman et al. (2006) has also been employed to quantify bioaccessibility of cadmium, chromium, copper, nickel, lead, and zinc to mute swans (*Cygnus olor*; Turner and Hambling 2012). The range finding study by Drexler (2013) found that at 2000 ppm in feed, MoS_2_ was 0 % bioaccessible whereas SMD was approximately 55 % bioaccessible. The 0 % bioaccessibility of MoS_2_ in feed may explain most of the reversible effects on growth and uptake seen between the two forms of Mo used in this study.

No treatment-related mortality occurred during this study; therefore, a median Lethal Concentration (LC_50_) was not calculated. Based on effects observed in proportional body weight change, the Lowest-Observed-Adverse-Effect Concentration (LOAEC) for bobwhite exposed to SMD was 2000 ppm in diet (253 mg Mo/kg body weight/day), and the NOAEC was 1200 ppm feed (134 mg Mo/kg body weight/day). The NOAEC for MoS_2_ was the highest dose tested (5000 ppm in diet, or approximately 545 mg Mo/kg body weight/day).

In addition to effects on growth, such as those described in the current study, ecological risk assessments frequently consider reproductive toxicity data (USEPA 2005). For example, Sample et al. (1996) developed no and lowest observed adverse effect levels (NOAELs and LOAEL) for Mo in birds of 3.5 mg/kg and 35.3 mg/kg/day, respectively, based on reproductive effects from SMD reported by Lepore and Miller (1965). Lepore and Miller (1965) fed breeding chickens the more soluble sodium molybdate (SMD) for 21 days. Similar to the results in the current study, these authors observed weight to be significantly reduced among adult birds consuming 2000 mg/kg Mo as SMD in the diet. In a subsequent experiment, Lepore and Miller (1965) reported that dietary Mo of 500 mg/kg Mo as SMD for 21 days reduced embryonic viability to zero. No other dietary concentrations were evaluated and published details of the study are lacking.

Results from Lepore and Miller (1965) were used by Sample et al. (1996) to develop wildlife screening levels because it represents the only study of effects of Mo on avian reproduction. However, Lepore and Miller (1965) contains multiple deficiencies such that uncertainty associated with effect thresholds derived from this study are high. Specific issues include, dose concentration data were not measured, but were nominal; reproductive effects were based on a single exposure dose; the study reports neither food intake rates nor body weights for test animals, requiring the use of assumed values to derive doses; details of study results and experimental protocols are lacking; etc. Further, Lepore and Miller (1965) only evaluated the more bioavailable form of Mo, SMD. The current study corrects many of these deficiencies. Although reproductive data for MoS_2_ are lacking, the results from the current study on bobwhite quail show that effects associated with this more environmentally prevalent and less bioavailable Mo form are much less than those observed for SMD. These MoS_2_ data should support more realistic representations of exposure and risks to avian receptors from environmental Mo.

## Conclusions

No treatment-related mortality occurred during this study; therefore, a median LC_50_ was not calculated. Based on effects observed in proportional body weight change, the LOAEC for northern bobwhite exposed to Mo as SMD was 2000 ppm in diet (253 mg Mo/kg body weight/day), with a NOAEC of 1200 ppm feed (134 mg Mo/kg body weight/day). The NOAEC for Mo as MoS_2_ was the highest dose tested (5000 ppm in diet, or approximately 545 mg Mo/kg body weight/day).

Recovery of SMD-treated birds was observed following a 5-day period during which the birds received untreated feed, indicating that the growth-related effects noted for SMD Mo exposure were reversible. The differences in growth-related effects and Mo uptake between SMD and the more environmentally relevant form of Mo, MoS_2_, could be due to bioavailability differences for these two forms of Mo. Furthermore, the reversible effect on growth observed for SMD (at dietary levels of 2000 and 3000 ppm) but not MoS_2_ also may be related to the presence of inorganic sulfate in the latter form. The bobwhite NOAEC for MoS_2_ established in this study can be used to better estimate the risks of exposure to ground dwelling avian species that may be present near Mo mining operations.

## Footnotes

1 Whereas USEPA (2007) defines bioavailability as the fraction of an ingested dose that crosses the gastrointestinal epithelium and becomes available for distribution to internal target tissues organs, bioaccessibility is defined as the fraction of the chemical extractable from its matrix (e.g., soil, sediment, food) into the gastrointestinal tract that is available for absorption (Ruby et al. 1996; NRC 2003; Koch and Reimer 2012). Given that bioavailability necessarily depends first on the solubility of the metal ion and further uptake of the soluble fraction, bioaccessibility can be considered as a conservative estimate of bioavailability for metals.
